# History of pharmacology: 1—the Department of Pharmacology of the University of Tartu (Dorpat): genealogy and biographies

**DOI:** 10.1007/s00210-022-02328-x

**Published:** 2022-11-21

**Authors:** Athineos Philippu, Roland Seifert

**Affiliations:** 1 Department of Pharmacology and Toxicology, Institute of Pharmacy, University of Innsbruck, Kranebitter Allee 26, 6020 Innsbruck, Austria; 2 Institute of Pharmacology, Hannover Medical School, Carl-Neuberg-Str. 1, 30625 Hannover, Germany

**Keywords:** Pharmacology, Tartu, Dorpat, History, Genealogy, Biographies

## Abstract

The purpose of this article is the historical survey of the foundation and development of pharmacology in Tartu (Dorpat), Estonia. Pharmacology was founded in Tartu by Naunyn, Buchheim, and Schmiedeberg. Genealogy and biographies including selected references of pharmacologists and pupils, who acted from the very beginning to today as directors of the Department of Pharmacology, as well as its successor, the Institute of Pharmacology and Toxicology, are presented and commented. This history also illustrates the conditions that are important for the development of new scientific areas. It is not a central geographical location or a formal “center of excellence” with lots of financial resources but rather brilliant researchers with the right spirit and vision and academic freedom. The implications of the early history of pharmacology for the future of science are discussed.

## Background

In 2023, *Naunyn–Schmiedeberg’s Archives of Pharmacology* celebrates its 150th anniversary. It is the oldest pharmacological journal. *Naunyn–Schmiedeberg’s Archives of Pharmacology* is the official journal of the Deutsche Gesellschaft für Experimentelle und Klinische Pharmakologie und Toxikologie (German Society for Experimental and Clinical Pharmacology and Toxicology). The journal is a general pharmacological journal and covers all areas of pharmacology and toxicology. On its 125th anniversary in 1998, Klaus Starke wrote an excellent account on the first 125 years of the history of this journal (Starke 1998).

On the 150th anniversary of the journal, in a series of papers, various aspects on the history of *Naunyn–Schmiedeberg’s Archives of Pharmacology* will be dealt with. This particular paper focuses on the origins of pharmacology. A second accompanying paper focuses on the subsequent development of pharmacology in Strasbourg (Philippu and Seifert 2023). Another paper covers the bibliographic development of the journal and its tremendous internationalization starting with the introduction of compulsory use of the English language in 1972 (Dats et al., 2023). Internationalization is highlighted by the close relationships of pharmacologists from Germany and Japan (Hattori et al. 2022; Hattori and Seifert, 2023). Future planned papers in *Naunyn–Schmiedeberg’s Archives of Pharmacology* will honor the publications of pharmacologists who were expatriated from Germany and Austria after 1933 and made great contributions to this journal (Löffelholz 2011). In this series of articles, the research topics in pharmacology in Germany from 1933 to 1945 will be analyzed and to which extent they served a military purpose. Honest efforts will be made to analyze the connection of the Nazi regime with pharmacology, and some hitherto unknown aspects have already been uncovered (Philippu and Seifert 2023).

When talking with colleagues, quite often you may hear voices that the name of the journal featuring the names of two researchers from the nineteenth century sounds very old-fashioned and is very hard to pronounce. And for this reason, it is probably a good idea not to submit a paper to this journal. This very commonly heard preconceived opinion could not be more distant from the truth and simply reflects ignorance of the history. Both Bernhard Naunyn and Osswald Schmiedeberg were nominees for the Nobel prize and outstanding role models, both as researchers and as mentors (Pohar and Hansson 2020). Osswald Schmiedeberg was nominated 18 times for the Nobel prize, and Bernhard Naunyn was nominated three times (Pohar and Hansson 2020). In addition, Bernhard Naunyn and Oswald Schmiedeberg, together with Rudolf Buchheim, were the very founders of pharmacology. The birth of pharmacology took place in Tartu (Dorpat), Estonia. To remind the international pharmacological community of its origins, in this article, we will trace the history of the Department of Pharmacology at the University of Tartu.

**Fig. 1:**
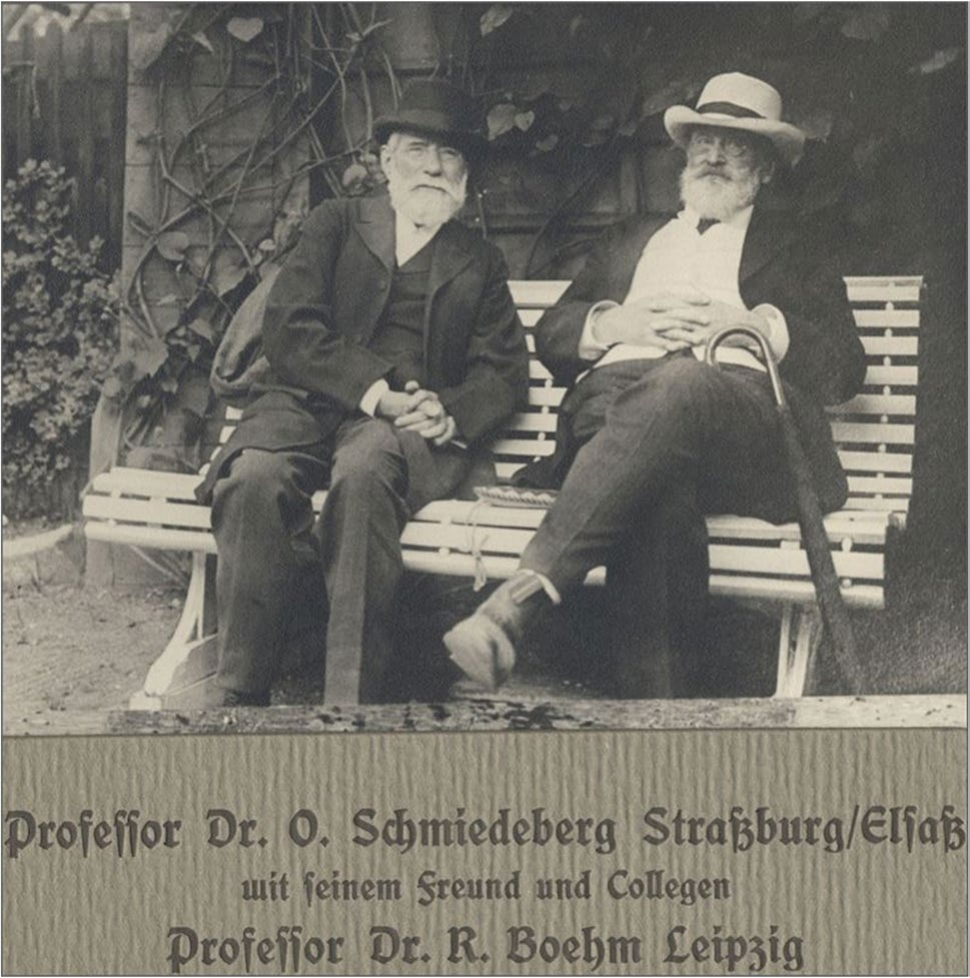
Oswald Schmiedeberg (Strasbourg/Alsace) with his friend and colleague Rudolf Boehm (Leipzig). The figure was taken about 1919 probably in Baden-Baden (Philippu 2007)

Figure 1 shows two eminent scholars from the Tartu genealogy of pharmacologists, Oswald Schmiedeberg and Rudolf Boehm. They were friends, and the photo shows *in pars pro toto* that tight personal relationships played a fundamental role in the beginnings of pharmacology.

## Genealogy

Years of directorship in parentheses, boldface written numbers refer to biographies. Pupils and their final positions indicated in italics.

### *Department of Pharmacology*

**Table:** 

**1. Berhard Naunyn**	
**2. Rudolf Richard Buchheim (1847–1867)**	*E. Heubel, professor of Pharmacology, Kiev (1876–1890)*
	*G. Gaehtgens, professor of Pharmacology, Giessen (1880–1890)*
	*O. Schmiedeberg, professor of Pharmacology, Strasbourg (1872–1918)*
**3. Oswald Schmiedeberg (1867–1872)**	*Selected pupils are presented in Philippu and Seifert (2023)*
**4. Rudolf Albert Martin Boehm (1872a,b–1881)**	*V. Podwyssotzky*
**5. Hans Horst Mayer (1882–1884)**	*S. Zaleski*
**6. Eduard Rudolf Kobert (1886–1897)**	*A. Grünfeld*
**7. Stanislav Czirwinski (1897–1902)**	
**8. David Lavrov (1902–1918)**	*M. Vilberg*
	*V. Vorontsov, professor, Voronezh*
	*F. Tjulpin, professor, Odessa*
	*E. Svolarovski, professor, Riga*
**9. Paul Trendelenburg (1918)**	
**10. Siegfried Walter Loewe (1921–1928)**	
**11. Georg Barkan (1928–1938)**	*Kingisepp, professor, Tartu*
**12. Georg Kingisepp (1938–1972)**	*L. Nurmand, professor, Tartu*
**13. Lembit Allikmets (1972–1992)**	*A. Zharkovsky, professor, Tartu*
*Institute of Pharmacology and Toxicology*	
**14. Lembit Allikmets (1992–2001)**	*A. Zharkovsky, professor, Tartu*
**15. Leo Nurmand (ext. Prof. 1992–1994)**	
**16. Aleksander Zharkovsky (since 2001)**	

## Biographies

### Department of Pharmacology

#### Bernhard Naunyn

**Fig. 2:**
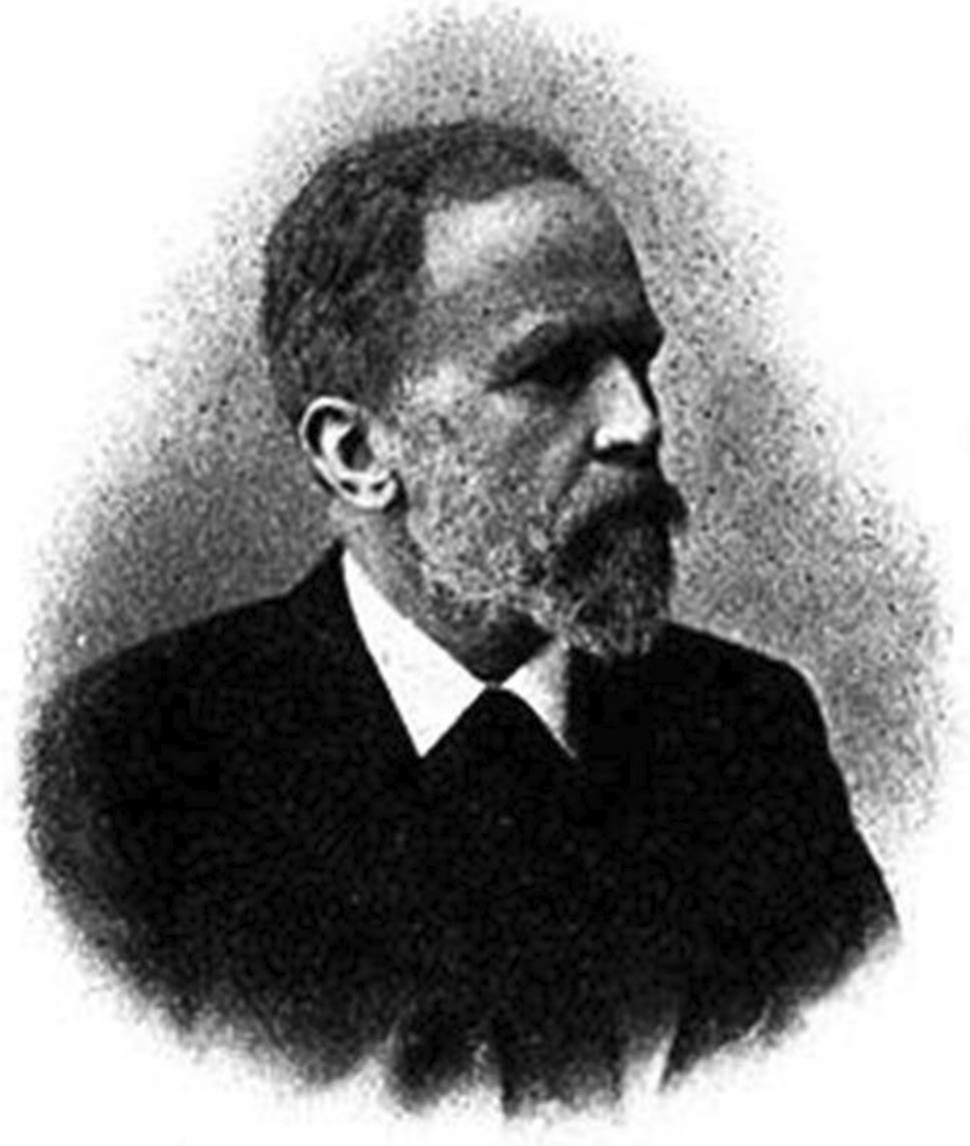
Bernhard Naunyn (Philippu 2007)

Bernhard Naunyn (Fig. 2) was born on 2 September 1839 in Berlin. Because of congenital hydrocephalus, he learned speaking only late. It is phenomenal that, despite of this severe disability, Naunyn, after studying law, physics, and chemistry, also studied medicine at the Friedrich-Wilhelms-Universität in Berlin. In 1863, he became a first assistant in the Medical Clinic of Charité. He worked mainly on diabetes mellitus (establishment of the dietary therapy of juvenile diabetes is his best-known scientific accomplishment) and habilitated 1867. After working temporarily as a physician in Berlin, in 1869, he was appointed professor of Special Pathology and Clinic at the University of Dorpat. In 1871, he moved to the University of Bern; in 1872/1872 to the (Kgl.) Albertus-Universität at Konigsberg, and in 1888, to the Kaiser-Wilhelms-Universität at Strasbourg.

During his stay in Konigsberg, in 1872, together with the pharmacologist Oswald Schmiedeberg (Strasbourg) and the pathologist and bacteriologist Erwin Klebs, he founded the *Archiv für experimentelle Pathologie und Pharmakologie* (now named *Naunyn–Schmiedeberg’s Archives of Pharmacology*), the first journal of pharmacology worldwide. The first issue of the journal appeared in 1873. In 1904, Bernhard Naunyn moved to Baden-Baden. He published several books about diseases such as diabetes and cholelithiasis. His collected work appeared in Würzburg (Naunyn 1909). He was nominated for the Nobel prize three times (Pohar and Hansson 2020). In Berlin-Kreuzberg, Naunyn is remembered with the Naunyn street.

Bernhard Naunyn died on 26 July 1925 in Baden-Baden.

#### Rudolf Richard Buchheim

**Fig. 3:**
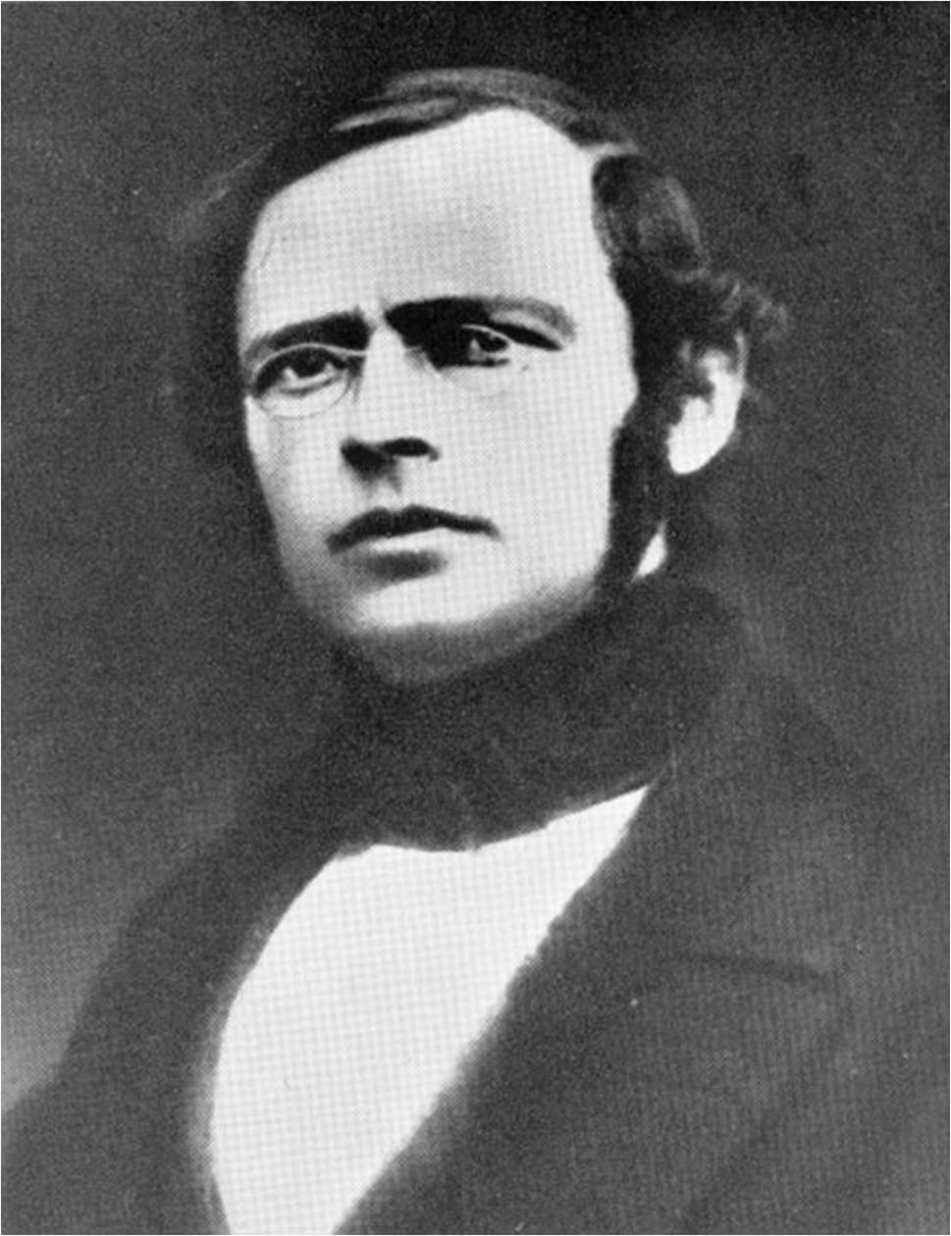
Rudolf Buchheim (Philippu 2007)

Rudolf Richard Buchheim (Fig. 3) was born on 1 March 1820 in Bautzen, Saxony. A biography of Buchheim has been presented recently (Philippu et al. 2022). In brief, in 1838, he studied medicine at the Academy of Surgical Medicine in Dresden and the University of Leipzig. In Leipzig, he was pupil of the professors Ernst Heinrich Weber (physiology and anatomy) and Karl Gotthelf (physiological chemistry). He received his medical degree by Gotthelf in 1845. Two years after his promotion, he was elected as an extraordinary professor and, in 1849, as a professor of Materia Medica, Dietetics, and History of Medicine at the University of Tartu (Dorpat).

Buchheim founded experimental pharmacology by introducing animal experiments and chemical analyses. For this purpose, he established the first laboratory of pharmacology worldwide in the basement of his private house, having at hand only small financial resources but many gifted students and positive scientific spirit. In his laboratory, medical students carried out pharmacological experiments on animals and chemical and biochemicals determinations for more than 20 years, until in 1860, he moved to the Department of Pharmacology at the Anatomical Theater (Theatrum Anatomicum) of the University of Tartu. During his stay in Tartu, about 90 (sic!) doctoral dissertations were accomplished; Oswald Schmiedeberg belonged to his most prominent pupils and doctoral candidates. Thus, Buchheim early on recognized that personal academic investment into gifted students (and not allocation of substantial financial resources) is the single most important factor for nurturing a scientific field.

The focus of the research in Tartu was the replacement of speculative drug properties by experimentally based knowledge; i.e., stringent logical thinking and analysis were implemented to the field of drugs. For example, lime water (1 part talk in 30 parts rainwater) had been used to treat tuberculosis by promoting acidification of the pulmonary foci. Buchheim postulated that due to the low water solubility, it is unlikely that enough talk is taken up into the organism so that lime water is of no use to treat tuberculosis. Since then, lime water or lime milk has been used to treat gastro-intestinal or dermal infections, but not anymore tuberculosis (Philippu et al. 2022).

To his most known publications belong those on endosmosis, importance of diffusion in inflammation processes, and effect of sodium sulfate (Buchheim 1853, 1854, 1855), His “Textbook of Pharmacology” (Lehrbuch der Arzneimittellehre) appeared in 1853–1856.

Buchheim was twice dean of the Medical Faculty of Tartu. In 1863, he rejected a call at the University of Breslau (Silesia). In 1866, he received offers for a professorship at the Universities of Giessen and Bonn. He accepted the offer of Giessen. However, in Giessen, he had again to establish a laboratory in his private house with very modest resources. In 1970, the Institut für Humanpharmakologie (Institute of Human Pharmacology) was renamed to Rudolf-Buchheim Institut für Pharmakologie. This was an initiative of the Director of the Institute of Pharmacology, Ernst Habermann (Schmidt et al. 2004).

Rudolf Richard Buchheim died of a heart disease followed by paralysis on 25 December 1879 in Giessen.

#### Oswald Schmiedeberg

**Fig. 4:**
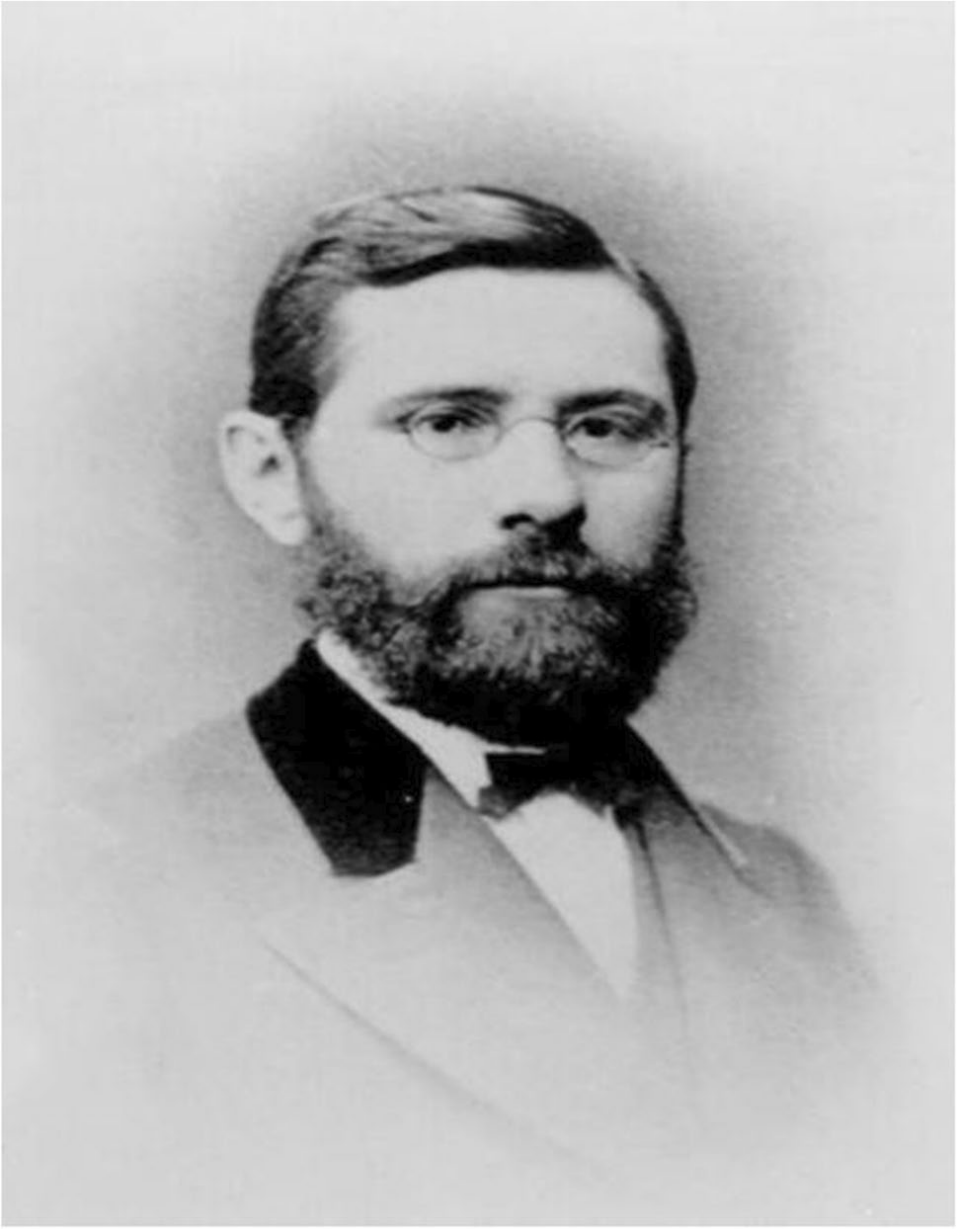
Johann Ernst Oswald Schmiedeberg (Philippu 2007)

Oswald Schmiedeberg (Fig. 4) was born on 11 October 1838 in the Russian Laidsen, Courland (today Latvia). He was son of a forester and spent his childhood in Estonia. Schmiedeberg, who spoke German with a slight accent, considered Estonia his home. He attended school in Tartu and studied medicine in the University. He was one of the most prominent doctoral students of R. Buchheim and he graduated in 1866. In the same year, he became an assistant of Buchheim. He habilitated in 1868 and, after R. Buchheim left to Giessen, he taught pharmacology and dietetics in Tartu.

One year later, he went to Leipzig to improve his experimental abilities with the physiologist Carl Ludwig using a kymograph invented by him and Gerhard Baltza at around 1840.

The first kymograph was developed in 1807 by the ophthalmologist and physicist Thomas Young. The Ruß-Kymograph (soot kymograph) of Ludwig and Baltza is the prototype for all devices developed since then used for the continuous registration of time-dependent biological data. The kymograph consists of a writer which scratches changes of, e.g., blood pressure on blackened glossy paper. The paper is fixed on a cylinder rotating with constant speed moved by a spring. The preparation of a uniformly blackened glossy paper demanded skillfulness. Many scientists, such as O. Schmiedeberg and R. Boehm, went to Ludwig to learn working with his kymograph. This kymograph was widely used, e.g., by v. Euler, Feldberg, Gaddum, and Holtz to discover neurotransmitters and their functions. Modern kymographs with electrically moving cylinders have been used in many laboratories of pharmacology and physiology until the 1960s.

In the laboratory of C. Ludwig, he met the pharmacologist Rudolf Boehm (see Background and Genealogy) to whom he was connected with many years of friendship (Fig. 1).

Back to Tartu, O. Schmiedeberg became first extraordinary professor of pharmacology, dietetics, and history of medicine and in 1871, as a successor of R. Buchheim, he became ordinary professor and director of the Department of Pharmacology. During the years he spent in Tartu and Leipzig, he published experimental findings concerning muscarine (Schmiedeberg and Koppe 1869), effects of drugs on the frog heart, and innervation of the dog heart (Schmiedeberg 1870, 1872).

In 1872, he was appointed professor at the Kaiser-Wilhelm-Universität and left to Strasbourg (Philippu and Seifert 2023). In the same year, together with E. Klebs and B. Naunyn, he founded the *Archiv für experimentelle Pathologie und Pharmakologie.* The first issue of the new journal appeared in 1873. Schmiedeberg was nominated for the Nobel prize 18 times (Pohar and Hansson 2020). Because of his eminence and importance for the international development of pharmacology, a separate paper will focus on Schmiedeberg and some of his many successful pupils (Philippu and Seifert 2023). Schmiedeberg followed and even surpassed Buchheim as mentor for the next generation of pharmacologists.

Oswald Schmiedeberg died on 12 July 1921 in Baden-Baden.

#### Rudolf Albert Martin Boehm

**Fig. 5:**
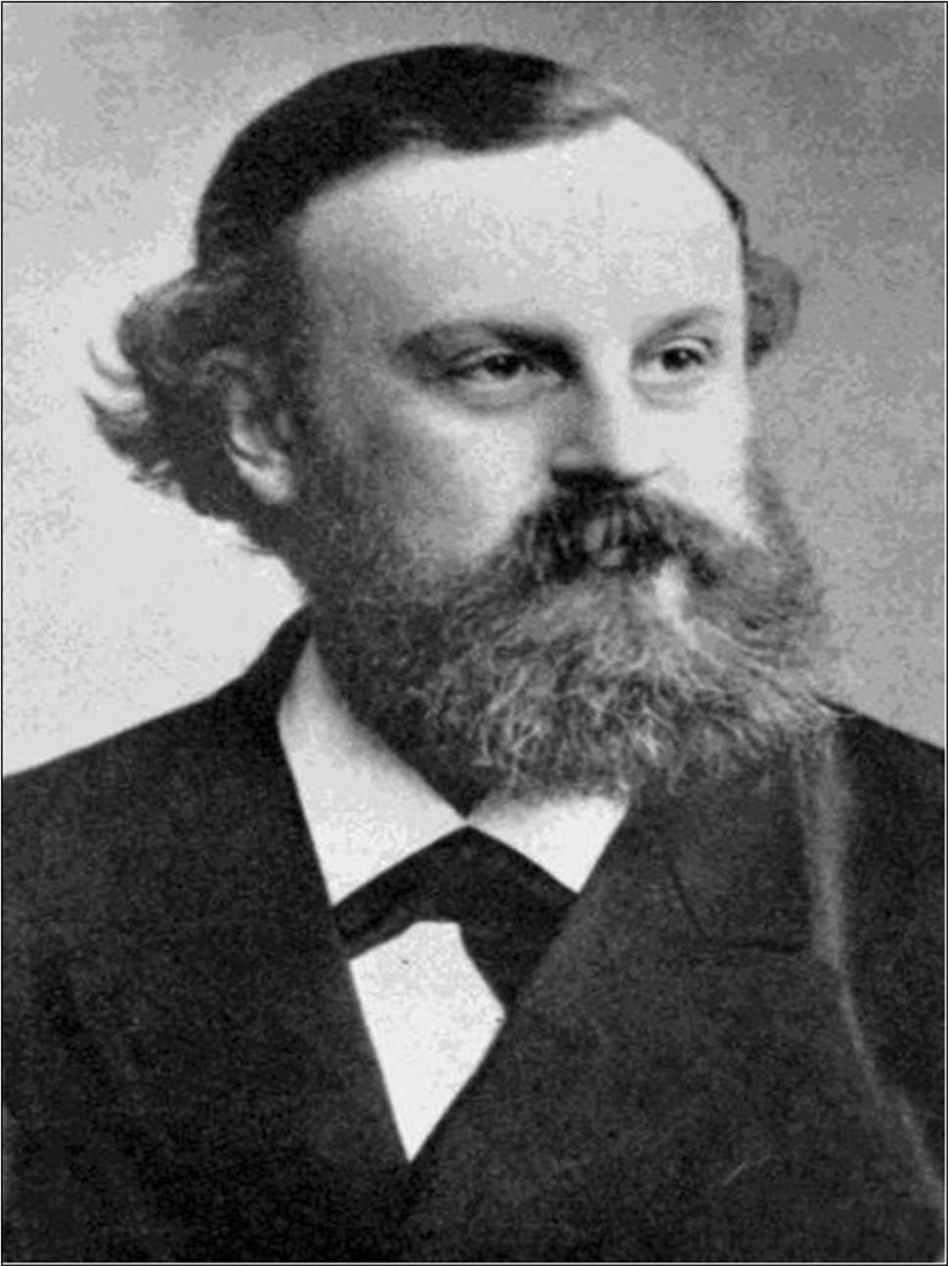
Rudolf Adolf Martin Boehm (Philippu 2007)

Rudolf Albert Martin Boehm (Fig. 5) was born in 1844 in Nördlingen, Bavaria. After studying medicine in Munich, Würzburg, and Leipzig, in 1867, he became Doctor of Medicine in Würzburg (Boehm 1868). After being an assistant at the Psychiatric Clinic of Würzburg for 2 years, in 1870, he went to Leipzig to work at the Institute of Physiology under the supervision of Carl Ludwig. In this Institute, where he met O. Schmiedeberg (see above), he started studying the pharmacological properties of various drugs including nicotine, aconitine, veratridine, and physostigmine on the frog heart. He continued and finished these studies as an assistant of Franz von Rinecker at the Juliusspital in Würzburg.

His research was interrupted by the German–French war, during which he served as physician of the Bavarian battalion. He obtained his habilitation in 1871 under A. Fick at the Institute of Physiology, University of Würzburg. In 1872, after O. Schmiedeberg left Dorpat to go to Strasbourg, R. Boehm was appointed extraordinary professor of pharmacology, dietetics, and history of medicine at the University of Dorpat, and one year later, he became ordinary professor.

His main research field was the investigation of the pharmacological and toxicological effects of substances occurring in plants, such as digitalis, muscarine, curare, veratrine, aconitine, and choline (Boehm 1872a, b; Boehm 1873; Boehm 1876a, b). During the stay in Dorpat, he published handbooks about intoxications (Boehm et al. 1876).

In 1881, Boehm left Dorpat for the University of Marburg to become a professor of pharmacology. Three years later, in 1884, he went to Leipzig to become director of the Institute of Pharmacology. In Leipzig, he continued his scientific work on alkaloids, curare (Boehm 1920), and antihelminthics and acted four times as dean of the medical faculty. Best known is his research on curare. He studied its pharmacological properties and divided the curare preparations into three types.

During his stay in Leipzig, the surgeon Arthur Läwen, who performed the first muscle relaxation, wrote: “A great disadvantage of superficial anesthesia is that the patient excessively tenses the abdominal muscles, especially when suturing the abdominal wall…. I used curarin, the active substance made by B o e h m from the curare preparations. Curarin has the great advantage over curare drugs of being a precisely dosed preparation, in which the same dose always corresponds to the same effect with absolute reliability. With the usual curare preparations, I would never have dared to experiment on humans. The effect of the abdominal wall suture (was) very clear and pleasant.”

R. Boehm retired in 1921. In 1963, under the directorship of Fritz Hauschild, the Institute of the University of Leipzig was renamed to Rudolf-Boehm-Institut für Pharmakologie und Toxikologie (Illes and Kästner 2004).

Rudolf Albert Martin Boehm died on 19 August 1926 in Bad Kohlburg, Lower Bavaria.

#### Hans Horst Meyer

**Fig. 6:**
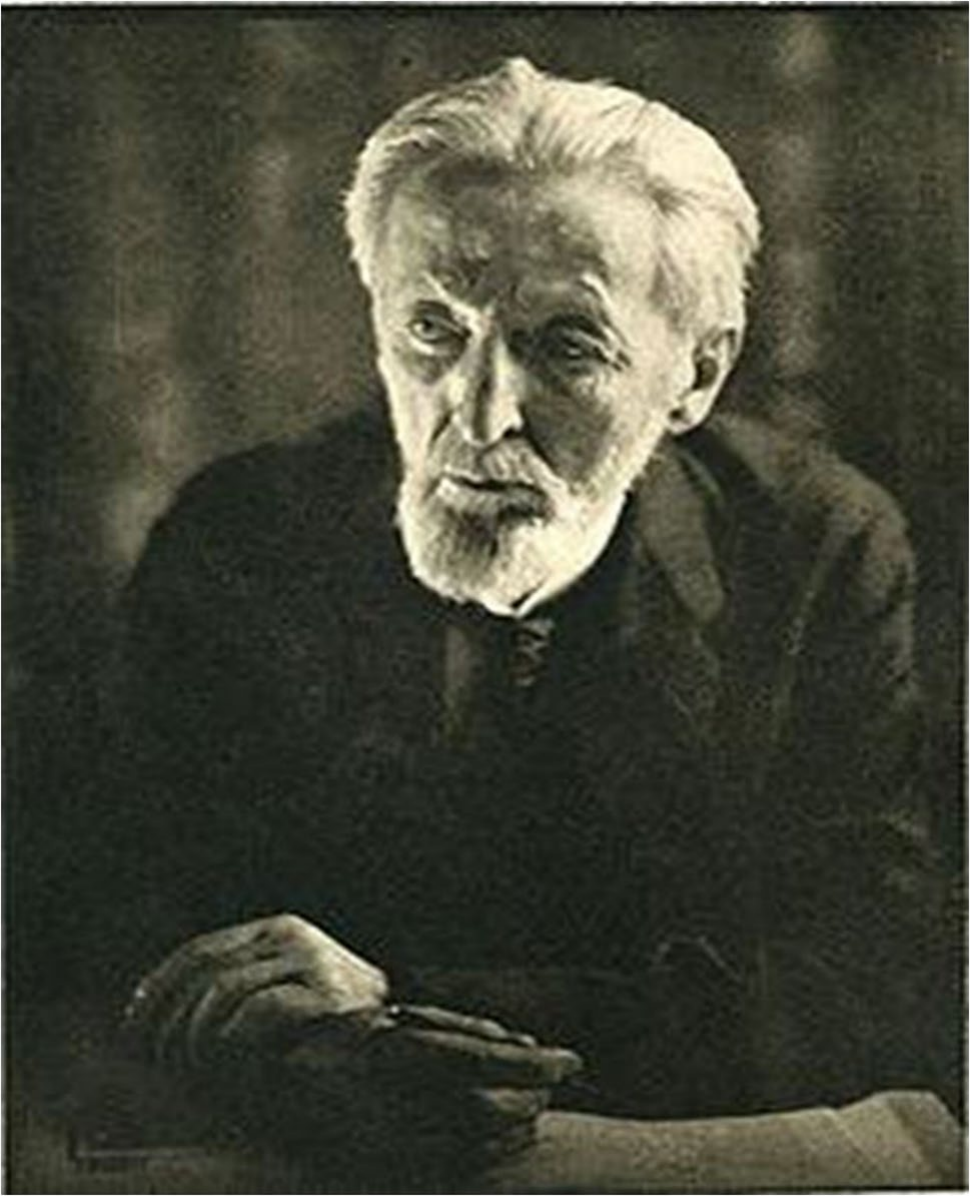
Hans Horst Meyer (Philippu 2007)

Hans Horst Meyer (Fig. 6) was born on 17 March 1853 in Insterburg, East Prussia. After his secondary school education in Insterburg and Konigsberg, he studied medicine in Leipzig, Berlin, and, again, in Konigsberg. He failed the state examination in pharmacology by Professor Max Jaffé, the director of the Institute of Pharmacology. Quite paradoxically, M. Jaffé proposed to Meyer to carry out his doctoral thesis at his Institute. Thus, M. Jaffé decisively influenced Meyers career. In 1877, H. H. Meyer became Doctor of Medicine. After his promotion, he went to the Institute of O. Schmiedeberg at Strasbourg (Philippu and Seifert, 2023). In 1879, together with O. Schmiedeberg, he investigated the presence of glucuronic acid in the urine and its detoxicating action.

In 1881, H. H. Meyer habilitated in pharmacology in Strasbourg (Meyer 1881). One year later, he was invited as extraordinary professor of pharmacology to Tartu University, and subsequently, he was appointed as director of the Department of Pharmacology, Dietetics, and History (successor of R. Boehm). During his short stay in Tartu, H. H. Meyer published seven experimental articles, among them determination of blood pH (Meyer 1883).

In 1884, H. H. Mayer was appointed as ordinary professor and head of the Institute of Pharmacology of the University of Marburg. His most known work concerns the lipid theory of narcosis (Meyer 1899, 1901). During his stay in Marburg, he was twice (1887 and 1894) dean of the Medical Faculty and rector of the University (1900–1901). In 1904, he was elected ordinary professor and head of the Institute of Pharmacology at the University of Vienna; he was retired as emeritus in 1924.

Hans Horst Mayer died on 6 October 1939 in Vienna.

#### Eduard Rudolf Kobert

**Fig. 7:**
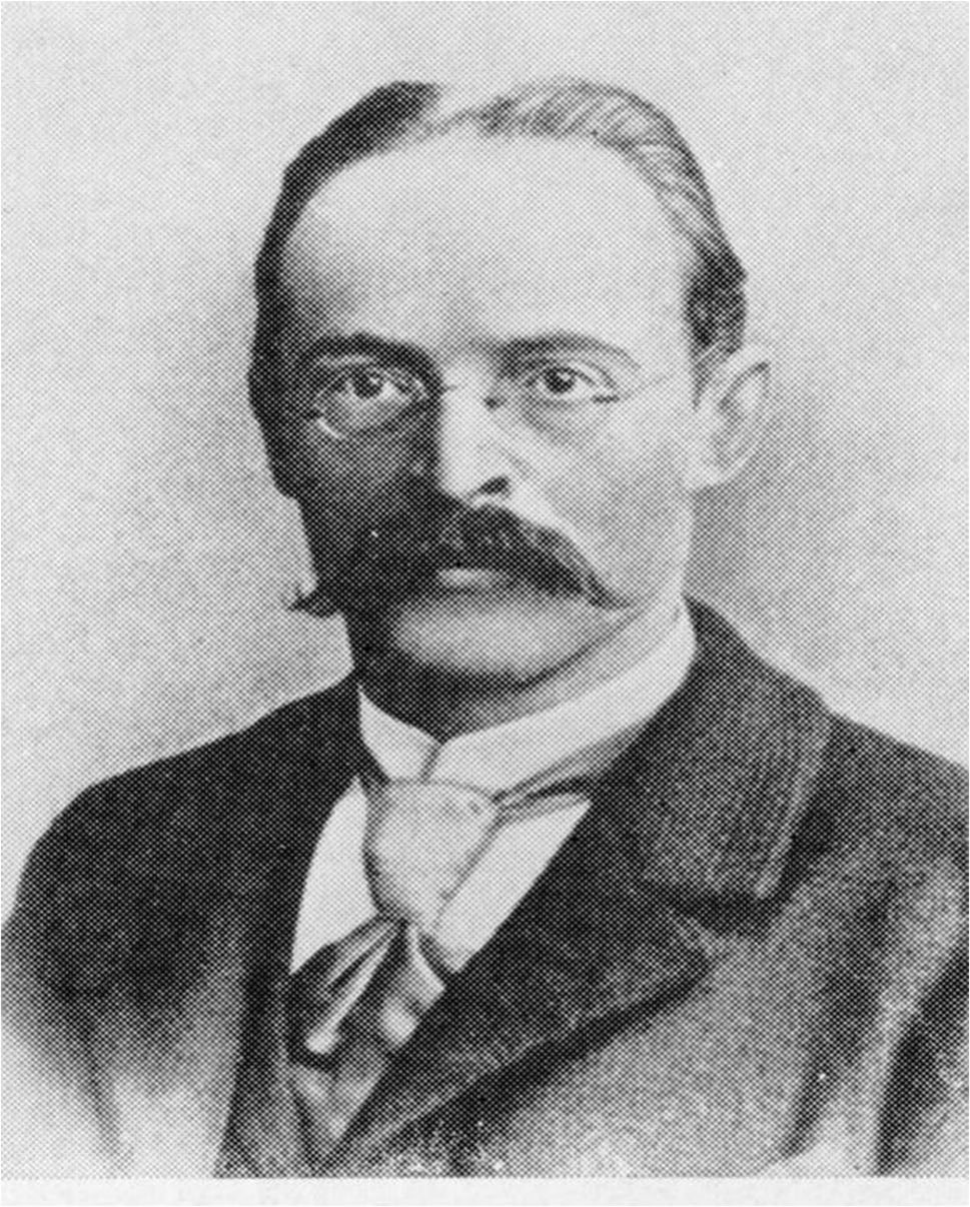
Eduard Rudolf Kobert (Philippu 2007)

Eduard Rudolf Kobert (Fig. 7) was born on 3 January 1854 in Bitterfeld, Saxony. His father died when he was 9 years old, and the orphan received his secondary education free of charge at the Franke’s educational institution. In 1873, he started studying medicine at the Halle University. In Halle, he became an assistant of the pharmacologist Hermann Köhler for 4 years, and in 1877, he defended his dissertation (Kobert 1877). Subsequently, Kobert went to Strasbourg (1880) and became assistant of O. Schmiedeberg for 6 years. In 1886, when he was 32 years old, he was appointed professor of pharmacology and director of the Department of Pharmacology, Dietetics, and History of Medicine at Tartu University, as successor of H. H. Meyer. In 1897, he had to return to Germany because of the Russification policy, and he worked for 2 years in Silesia as director of the Brehmer’s lung clinic. In 1899, he moved to Rostock as appointed professor of pharmacology at the Chair of Pharmacology, Physiological Chemistry, and Pharmacognosy as successor of Professor Otto Nasse. In 1906, he was elected rector of the University. In 1901 and 1910, he was dean of the Medical Faculty. Kobert published numerous experimental works and books on melanin (1901), agglutinins (Kobert 1909), saponins (Kobert 1914), and intoxications (Kobert 1902–1906).

Eduard Rudolf Kobert died on 27 December 1918 in Rostock.

#### Stanislav Czirwinski

**Fig. 8:**
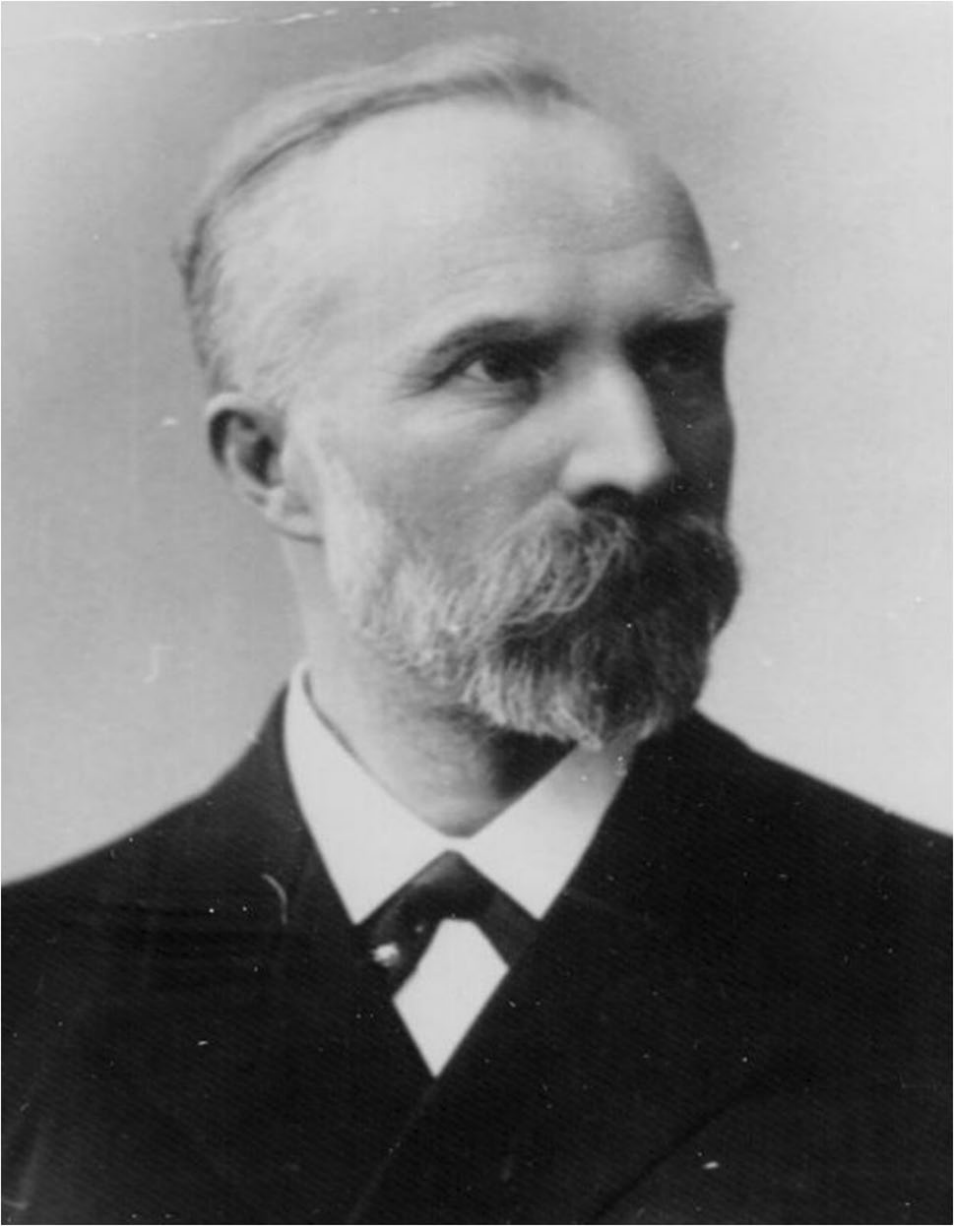
Stanislaw Czirwinsky (Philippu 2007)

Stanislav Czirwinski (Fig. 8) was born in 1852 in Mariampol, Lithuania. After finishing the secondary school education, he studied medicine at the University of Warsaw and graduated at the University of Moscow in 1875. During the Russian-Turkish war, he worked in several hospitals. From 1883 to 1884, he worked in Strasbourg with O. Schmiedeberg. In 1884, he returned to Moscow and in 1886, he became an assistant at the Department of Pharmacology. After defending his dissertation (Czirwinski 1891), he became Privatdozent. In 1897, he was appointed as professor and, as successor of R. Kobert, director of the Department of Pharmacology, Dietetics, and History of Medicine at the University of Tartu. S. Czirwinski worked mainly on vasomotor control and lymph. Most of his publications appeared before 1897.

Stanislav Czirwinski died in 1922 in Russia.

#### David Lavrov

**Fig. 9:**
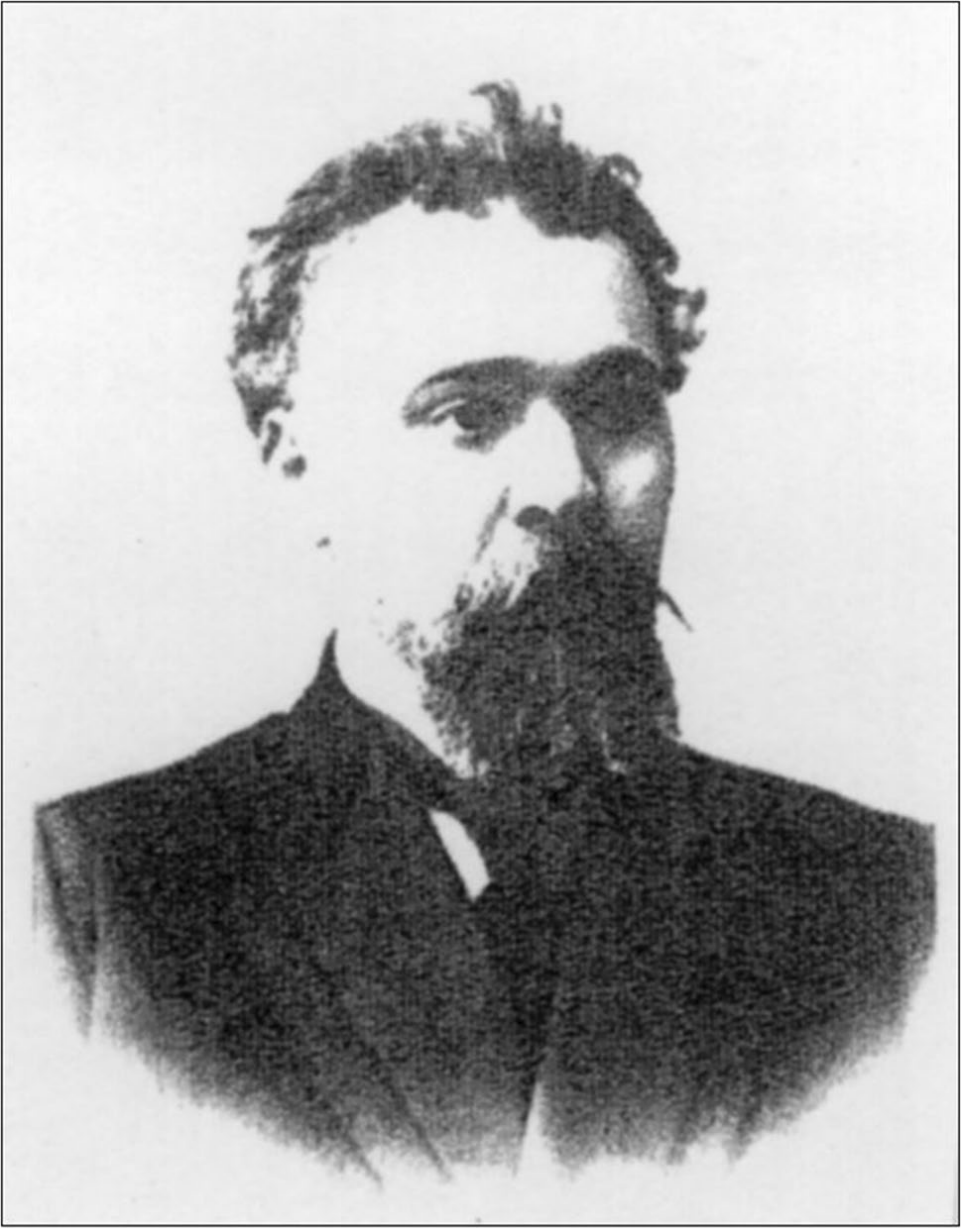
David Lavrov (Philippu 2007)

David Lavrov (Fig. 9) was born in 1897 in Yelets, Russia. As Privatdozent, D. Lavrov worked at the St. Petersburg Academy of Military Medicine (Russia) before, in 1912, he was appointed as director of the Department of Pharmacology, University of Tartu, as successor of S. Czirwinski. D. Lavrov was dean of the Faculty of Medicine from 1909 to 1910. In 1918, he left Tartu for Voronezh as director of the Department of Pharmacology. Thereafter, he worked in medical institutes in Odessa. During his stay in Tartu, D. Lavrov carried our research concerning comparative pharmacology and the effects of lecitin on isolated organs. He published almost exclusively in Russian language.

David Lavrov died in 1929 in Odessa.

#### Paul Trendelenburg

**Fig. 10:**
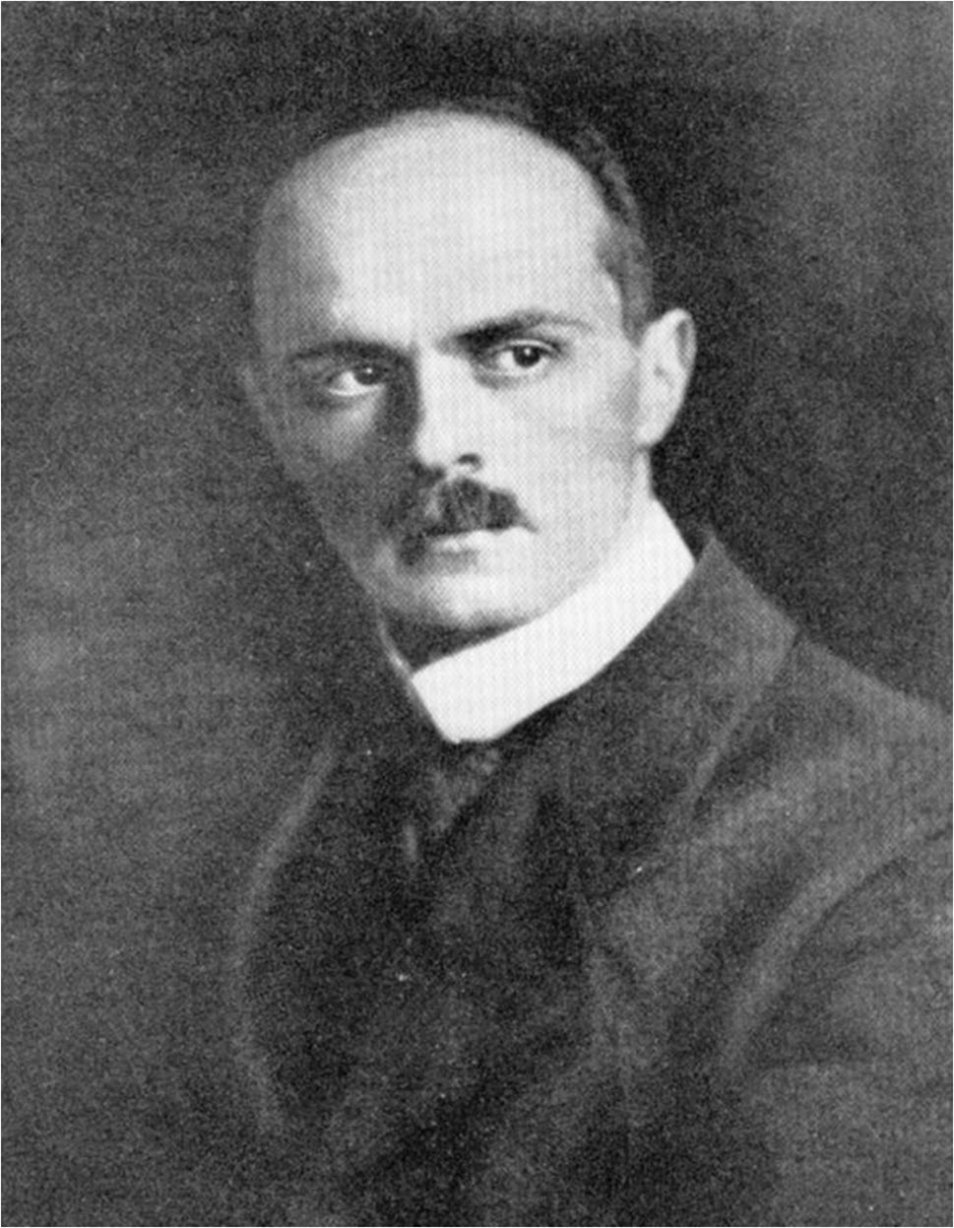
Paul Trendelenburg (Philippu 2007)

Paul Trendelenburg (Fig. 10) was born on 24 March 1884 in Bonn. In 1903, he started studying medicine at the universities of Grenoble and Leipzig and in 1908, he graduated at the Freiburg (Breisgau) University. He worked in Freiburg from 1909 to 1918 as an assistant in the pharmacological institute under the direction of Schmiedeberg’s student Walter Straub, and at the surgical clinic. In 1909, he defended his doctoral thesis (Trendelenburg 1909) and in 1912, he received his habilitation in pharmacology and toxicology. From 1916 onwards, he was an associate professor. In 1918, he was appointed as director of the Department of Pharmacology at Tartu, but his stay there, during the occupation of Estonia by the Germans, was very short (15 September to 18 December). Because of the arrival of Russian troops, he had to leave for Freiburg. Later, he was elected as director of the pharmacological institute in Rostock (1919–1923), Freiburg (1923–1927), and Berlin (1927–1930). His main research concerned adrenaline and the development of biological measurement procedures for the standardization of hormone preparations (Trendelenburg 1910).

Paul Trendelenburg, the father of the pharmacologist Ullrich Trendelenburg (Starke 1998), died on 4 February 1931 in Berlin, at the young age of 46 years.

#### Siegfried Walter Loewe

**Fig. 11:**
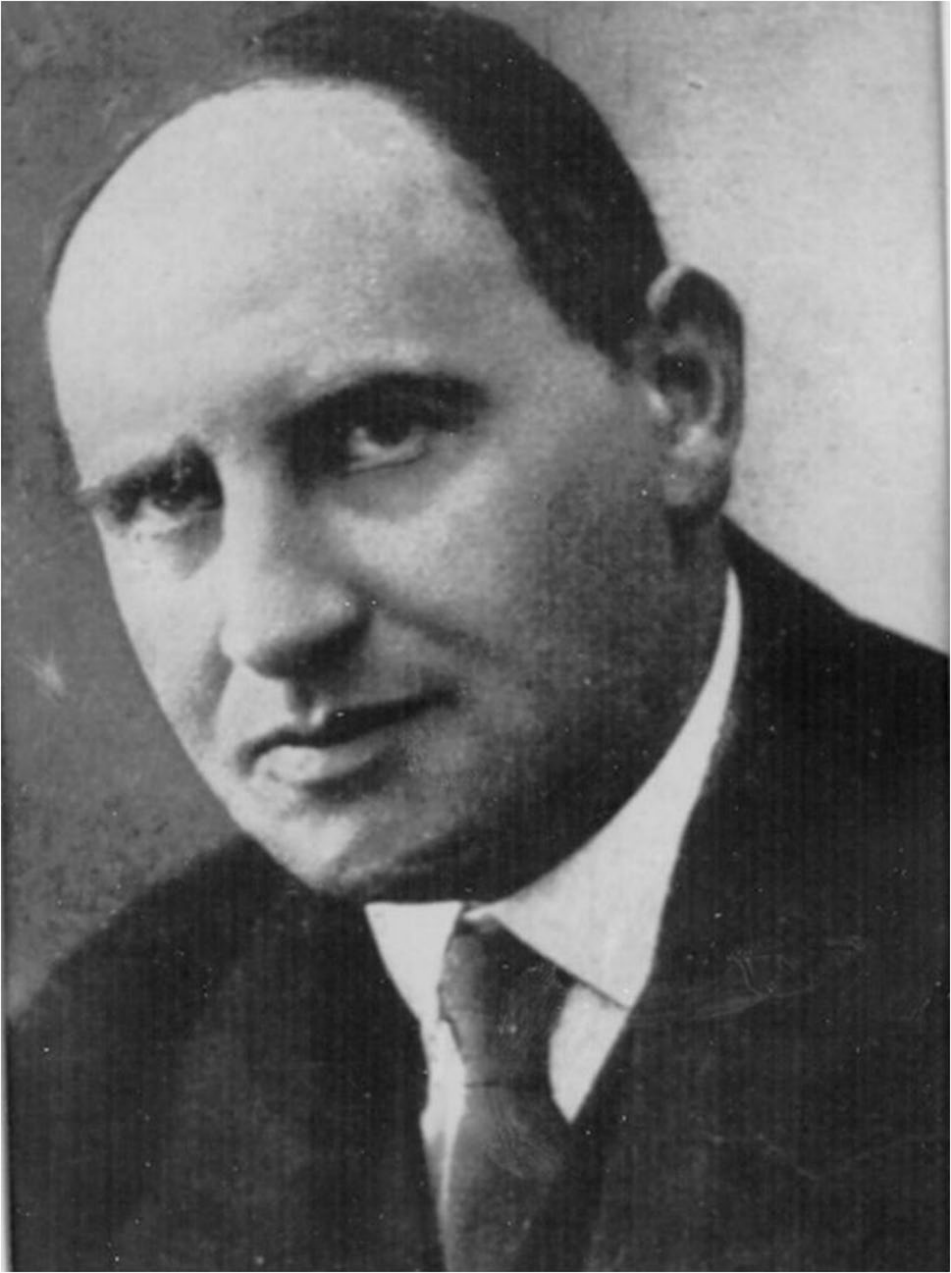
Walter Loewe (Philippu 2007)

Siegfried Walter Loewe (Fig. 11) was born on 19 August 1884 in Fürth, Bavaria. He received his secondary school education in Frankfurt/Main from 1893 to 1902 and studied medicine in the universities of Freiburg, Berlin, Strasbourg, and Munich. In 1905, he started working at the Institute of Physiological Chemistry, University of Strasbourg, and in 1908 he was awarded his doctoral degree (Loewe 1908). From 1910 to 1912, he worked at the University of Leipzig (chemical laboratory of the psychiatric clinic and Institute of Physical Chemistry). In 1912, he was as assistant at the Institute of Pharmacology, University of Göttingen, under Professor Wolfgang Heubner, a student of O. Schmiedeberg. In 1913, he habilitated for Pharmacology and from 1915 to 1918, as associate professor of pharmacology, he was appointed as director of the Institute. In 1918, he became an extraordinary professor.

From 1921 to 1928, S. Loewe was the director of the Department of Pharmacology, Dietetics, and History of Medicine at the University of Tartu. His research on sex hormones began in Tartu (Loewe 1926 a; Loewe et al. 1926). He also worked on theoretical and experimental problems of pharmacology (Loewe and Die 1927). In 1928, he returned to Germany as the successor of professor Lesser, head of the laboratory of the hospital at Mannheim, and thereafter, as honorary professor of the Ruprechts-Karls-University of Heidelberg. In 1933, because of his Jewish origin, he had to leave Germany and went to the USA. From 1935 to 1946, he worked as a researcher at the Institute of Pharmacology of Cornell University in New York. In 1946, he became research professor of pharmacology at the Institute of Pharmacology of Utah, Salt Lake City. Louis Goodman was the director of the institute. In the USA, S. Loewe continued his work on sex hormones and cannabis.

Siegfried Walter Loewe died on 24 August 1963 in Salt Lake City.

#### Georg Barkan

**Fig. 12:**
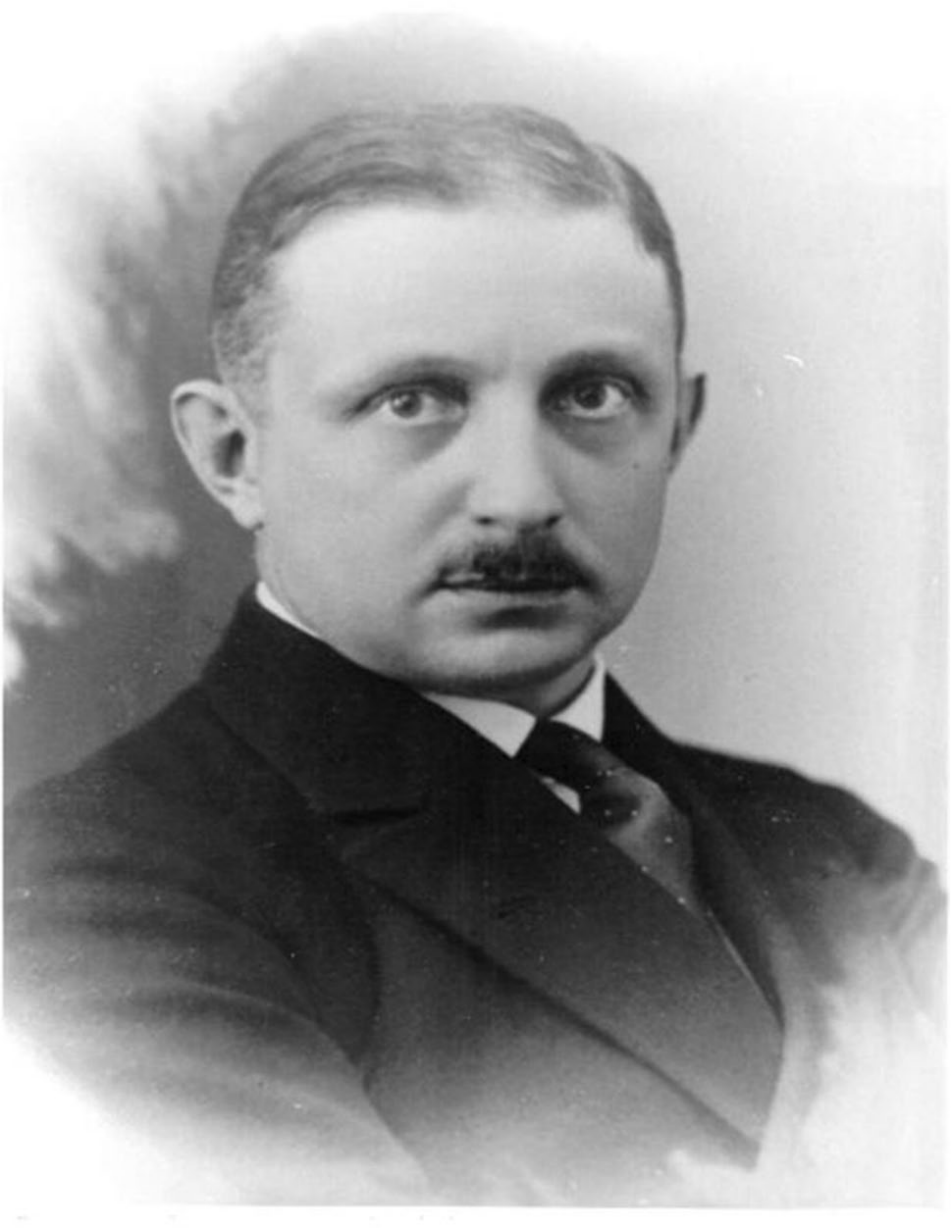
Georg Barkan (Philippu 2007)

Georg Barkan (Fig. 12) was born on 22 March 1889 in Polotzk, Belarus. After finishing his secondary school education in Breslau, he studied medicine in Freiburg, Breslau, and Munich. In 1914, he obtained his academic degree in Munich (Barkan 1914). During World War I, he was a surgeon in the German army. After the war, he worked for 3 years in Munich at the Institute of Physiology as an assistant lecturer. In 1919, he worked as a doctor in the Medical Clinic, University of Würzburg, and as an assistant in the Institute of Physiology, University of Munich. In 1923, he went to Alexander Ellinger at the Institute of Pharmacology in Frankfurt/Main. In 1927 he became Privatdozent and professor.

In 1929, G. Barkan was appointed as director of the Department of Pharmacology, Dietetics, and History of Medicine at the University of Tartu, as successor of S. W. Loewe. In 1936, the Faculty of Medicine of Tartu decided to fill the Department of Pharmacology with an Estonian although the Department would become vacant in 1937. His successor became G. Kingisepp. In 1937, Barkan had to go back to Germany. However, because of the law to restore the civil service, he had no career opportunities. In 1938, he left Germany and emigrated to the USA as an associate professor of biochemistry at the Boston Medical University.

During his stay in Tartu, the main scientific fields of G. Barkan and his coworkers were hemoglobin (Barkan and Schales 1936), metabolism of iron (Barkan and Schales 1936, 1937), and pharmacology of iodine (Barkan and Prik 1930; Barkan and Kingisepp 1932).

Georg Barkan died on 7 March 1945 in Boston.

#### Georg Kingisepp

**Fig. 13:**
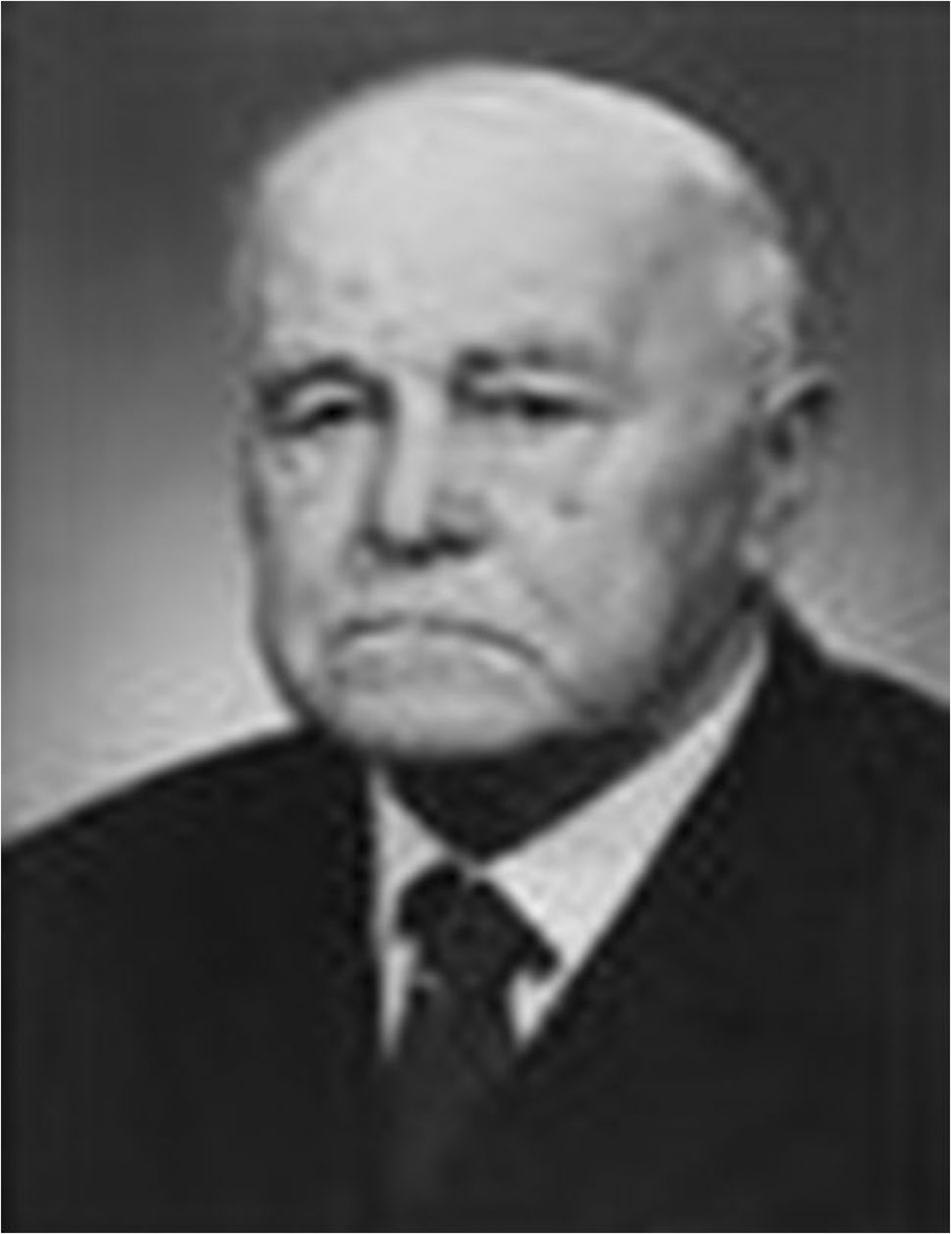
Georg Kingisepp (geni.com, 2021)

Georg Kingisepp (Fig. 13) was born on 30 May 1898 in Kurla, Kabala commune, Estonia. He studied at Retla village school and Allinku elementary school before entering Rakvere Teachers Seminary. In 1917, he graduated. But instead of working as a teacher, he became a clerk at Türi paper and cardboard factory. After getting the certificate of secondary education in 1921, he started studying medicine at the University of Heidelberg. After interrupting his studies because of financial reasons, he graduated in 1927, and one year later, he completed his dissertation in Heidelberg (Kingiseep 1928). From 1929 to 1930, he worked as a teacher in Särevere, Estonia.

In 1930, G. Kingisepp became a junior assistant at the Department of Pharmacology, Dietetics, and History of Medicine at Tartu under G. Barkan. After spending one-year on a scholarship at the Institute of Pharmacology in Edinburgh under the guidance of professor A. J. Clark, in 1937, he worked again on a scholarship at the Institute of Pharmacology, University of Münster, under professor L. Lendle. In the same year, he got the venia legend and in 1938, he was elected as director of the Department of Pharmacology, Dietetics, and History of Medicine at Tartu University.

As head of the Department of Pharmacology, G. Kingisepp and his workers published almost exclusively in Estonian language. He retired in 1972.

Georg Kingisepp died on 19 August 1974 in Tartu.

### Institute of Pharmacology and Toxicology

In 1992, the Department of Pharmacology, Dietetics, and History of Medicine was reconstructed and renamed to Institute of Pharmacology and Toxicology. The Institute has various departments which are not considered in this historical survey.

## Lembit Allikmets

**Fig. 14:**
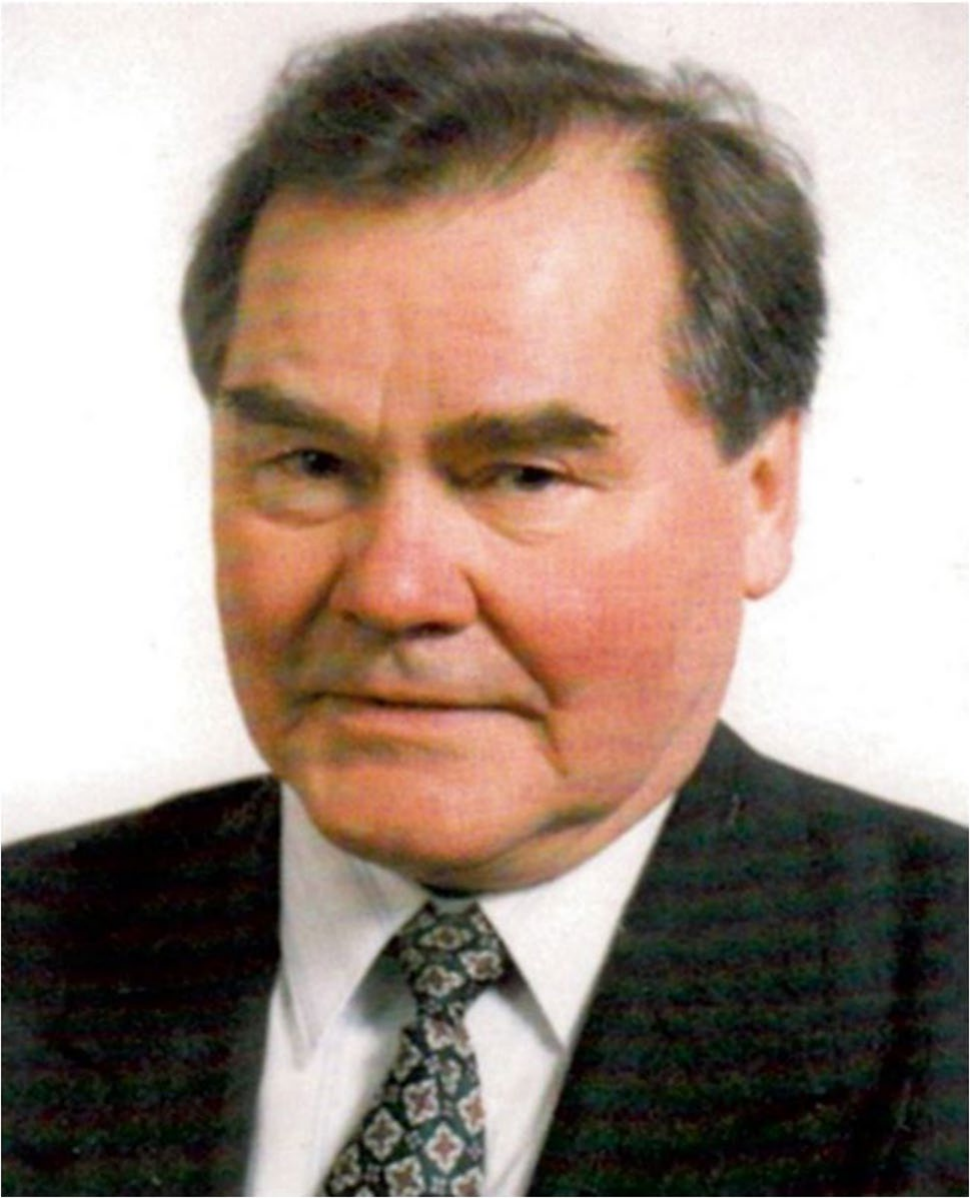
Lembit Allikmets (Toomsalu 2016)

Lembit Allikmets (Fig. 14) was born on 18 June 1936 in Allika village, Estonia. In 1954, he graduated from the Tallinn Medical Secondary School and started studying medicine at the University of Tartu. He graduated in 1960 and started working in pharmacology at the Institute of Experimental Medicine in St. Petersburg.

From 1963 to 1965, L. Allikmets worked as a research fellow in the central Medical Research Laboratories, University of Tartu, and from 1966 to 1972, as a senior research fellow and as director of the Division of Experimental Pathology and Pharmacology. He completed his dissertation (Allikmets 1970), and in 1972, he became first professor and head of the Department of Pharmacology at Tartu University and 1992 head of the newly constructed Institute of Pharmacology and Toxicology. He was elected twice as dean of the Medical Faculty. From 1966 to 1967 and in 1990, he worked as scholar at Yale University, from 1978 to 1979 at New York and Wisconsin Universities.

L. Allikmets published several pharmacological textbooks in Estonian language. His research mainly deals with mechanism of action of psychotropic drugs and neurotransmitters such as serotonin and GABA (Vasar et al. 1986; Rudissaar et al. 1999), as well as with pharmacokinetics (Rägo et al. 1986).

L. Allikmets is a professor emeritus since 2001.

## Leo Nurmand

**Fig. 15:**
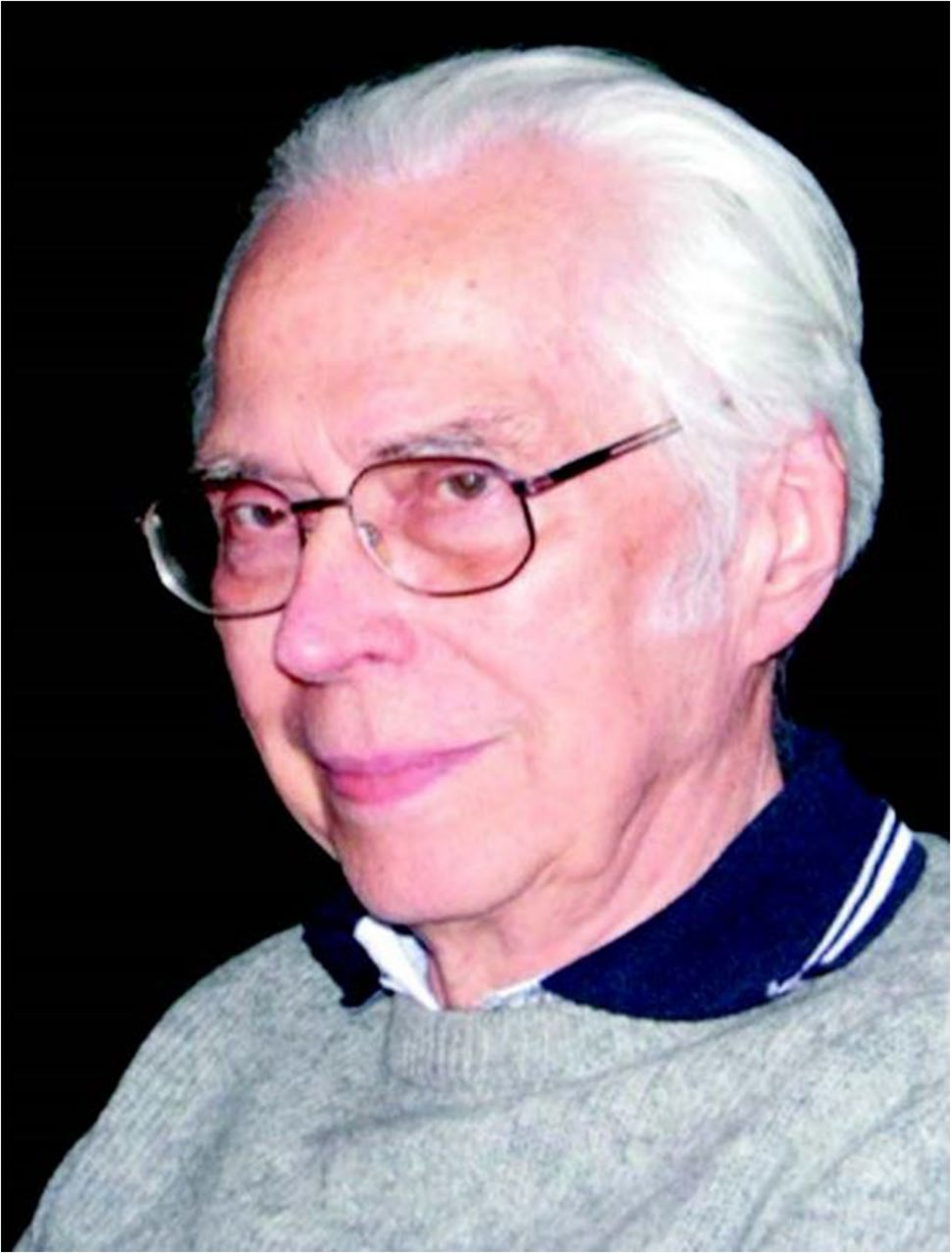
Leo Nurmand (Anomynous 2016)

Leo Nurmand (Fig. 15) was born on 12 June 1929 in Tallinn, Estonia. In 1947, he started studying medicine at the Tartu University. He graduated in 1953 and worked as a teaching assistant at the Department of Pharmacology under professor G. Kingisepp. From 1965 to 1971, he was senior lecturer, from 1971 to 1976 associate professor, and in 1975, he became Doctor of Medicine (Nurmand 1975).

From 1976 to 1992, L. Nurmand was professor, and from 1992 to 1994, extraordinary profesor at the Institute of Pharmacology, University of Tartu. He became emeritus in 1994.

Leo Nurmand’s fields of research were mechanisms of action of drugs and pharmacokinetics, sedatives, analgesics, and tranquilizers. He published mainly in Estonian and Russian language.

Leo Nurmand died on 13 May 2016 in Tartu.

## Aleksander Zharkovsky

**Fig. 16:**
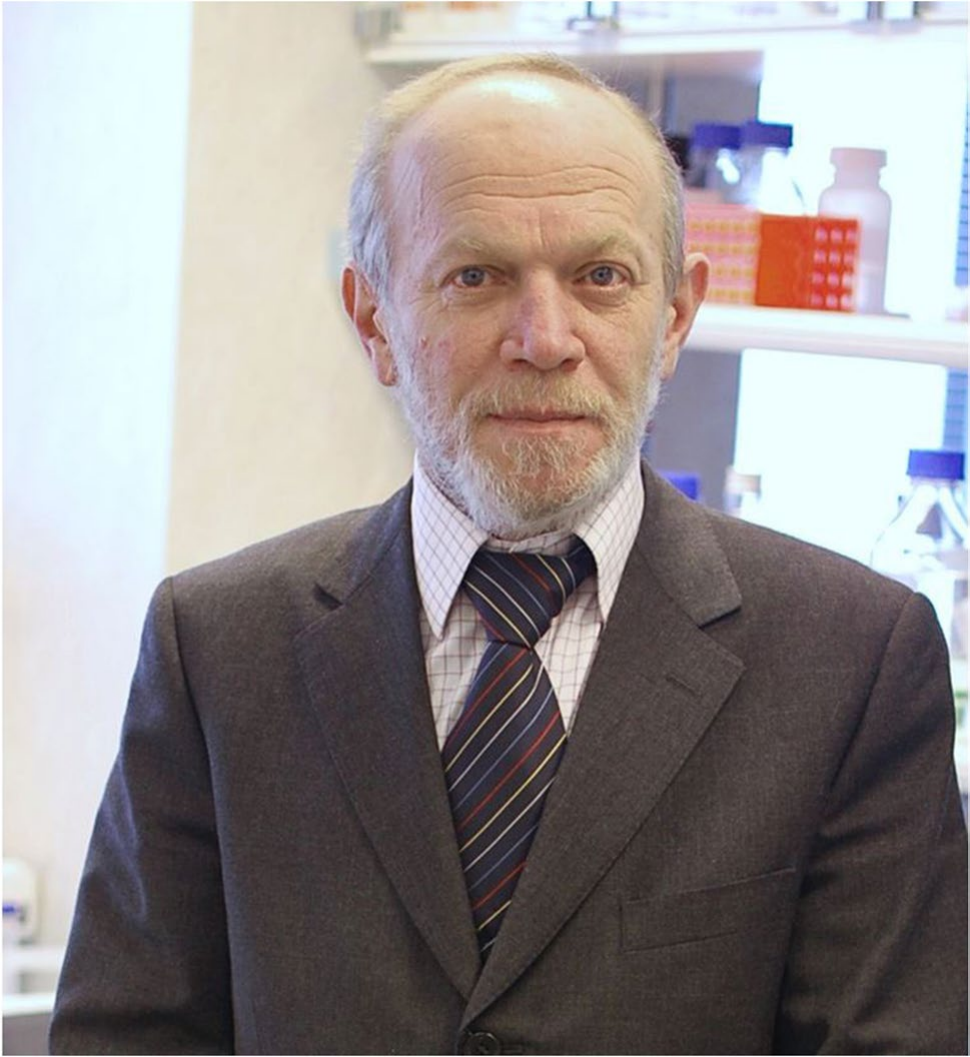
Aleksander Zharkovsky (A. Zharkovsky: researchgate.net/profile, 2021)

Aleksander Zharkovsky (Fig. 16) was born on 31 January 1950 in Gomel, Belarus. After studying medicine at the University of Tartu, he was a post-graduate student at the Department of Pharmacology from 1974 to 1978, thereafter a research fellow, a lecturer, a senior lecturer, and an associate professor (1978 to 1990). In 1988, he defended his dissertation at Leningrad Institute of Experimental Medicine (Zharkovsky 1988). In 1989, he became professor and in 1993, an ordinary professor and head of the chair of Pharmacotherapy and Toxicology. In 2001, A. Zharkovsky became head of the Institute after the retirement of L. Allikmets.

The main research fields of A. Zharkovsky and his coworkers are addiction (Zharkovsky et al. 1999) as well as neuronal death, neurodegenerative processes, and diseases (Kaasik et al. 2001; Zharkovsky et al. 2003; Heidnets et al. 2006; Somelar et al. 2021).

## Concluding remarks

It is quite astonishing that the origins of pharmacology were not laid in one of the large central European universities but rather in the periphery. Obviously, this peripheral, remote location in Estonia and the mingling of local (rather rural) influences with cultural influences from Russia and Germany provided the optimal breeding ground for the birth of a new discipline that, by its nature, is interdisciplinary and does not fit the traditional canon of medical specialties. Thus, the absence of suffocating academic traditions and preconceived opinions often found in traditional “big” universities obviously facilitated the birth of pharmacology.

The founders of *Naunyn–Schmiedeberg’s Archives of Pharmacology* were aware of the interdisciplinary nature of the new field, being a pharmacologist (Schmiedeberg), a clinician (Naunyn), and a pathologist (Klebs). This fundamental concept of pharmacology has proven to be valid until today (Dats et al. 2023).

It is also impressive that the Department of Pharmacology of Tartu was an attractive destination for so many international pharmacologists during the first 50–60 years of its existence despite the rather peripheral location in Europe. This lends support to the notion that the academic atmosphere in Tartu must have been very productive for the young discipline without official status as a research center of excellence.

Strong personalities, nurturing young scientists, and cultural exchange were more important for the development of pharmacology than monetary resources. New fields of science can even be founded in a private home. The case study of the origins of pharmacology is very relevant for the future development of science.

The general contemporary assumption is that large financial resources are a “must-have” for scientific success. What we can learn from Buchheim is that time and resources are better invested into the training of talented young scientists rather than writing many (most often non-funded) grant proposals and aiming for a high ranking in excellence tables, summing up impact factors, number of publications, and the amount of third-party funding. In times when financial resources for the entire research community again become limited because “Excellence” clusters consume a big share, we must rethink how we develop science by looking back into the history. For many projects, it simply takes creativity and collaboration with gifted young people. Like pharmacology, many successful Silicon valley businesses started in private homes.

Obviously, close human relationships, friendship, mutual respect, and trust (illustrated in Fig. 1) rather than fierce academic competition for grant money, personnel, and journal impact factors played a great role during the initial phase of the existence of pharmacology and whether a pharmacologist would accept an offer to go to Tartu. The Department of Pharmacology of the University of Tartu also turned out to be an outstanding catalyst for global academic careers (Philippu and Seifert 2023). In conclusion, for pharmacology, Tartu was the Stanford University and Harvard University of the nineteenth century.

## Data Availability

All relevant literature is cited. Sources of photographs are provided.
